# Concentration-Dependent Dual Mode of Zn Action at Serotonin 5-HT1A Receptors: In Vitro and In Vivo Studies

**DOI:** 10.1007/s12035-015-9586-3

**Published:** 2015-12-12

**Authors:** Grzegorz Satała, Beata Duszyńska, Katarzyna Stachowicz, Anna Rafalo, Bartlomiej Pochwat, Christine Luckhart, Paul R. Albert, Mireille Daigle, Kenji F. Tanaka, René Hen, Tomasz Lenda, Gabriel Nowak, Andrzej J. Bojarski, Bernadeta Szewczyk

**Affiliations:** 1 Institute of Pharmacology Polish Academy of Sciences , Smetna 12, PL 31-343 Krakow, Poland; 2 Ottawa Hospital Research Institute, UOttawa Brain and Mind Research Institute , 451 Smyth Road #2464, Ottawa, ON K1H-8M5 Canada; 3 Department of Neuropsychiatry, School of Medicine, Keio University, Tokyo, 160-8582 Japan; 4 Department of Psychiatry, Columbia University Medical Center and Research Foundation for Mental Hygiene, New York State Psychiatric Institute, New York, NY 10032 USA

**Keywords:** Zn, Serotonin, 5-HT_1A_, Autoreceptor, Depression, Binding, Behavioural studies

## Abstract

Recent data has indicated that Zn can modulate serotonergic function through the 5-HT_1A_ receptor (5-HT_1A_R); however, the exact mechanisms are unknown. In the present studies, radioligand binding assays and behavioural approaches were used to characterize the pharmacological profile of Zn at 5-HT_1A_Rs in more detail. The influence of Zn on agonist binding to 5-HT_1A_Rs stably expressed in HEK293 cells was investigated by in vitro radioligand binding methods using the agonist [^3^H]-8-OH-DPAT. The in vivo effects of Zn were compared with those of 8-OH-DPAT in hypothermia, lower lip retraction (LLR), 5-HT behavioural syndrome and the forced swim (FST) tests. In the in vitro studies, biphasic effects, which involved allosteric potentiation of agonist binding at sub-micromolar Zn concentrations and inhibition at sub-millimolar Zn concentrations, were found. The in vivo studies showed that Zn did not induce LLR or elements of 5-HT behavioural syndrome but blocked such effects induced by 8-OH-DPAT. Zn decreased body temperature in rats and mice; however, Zn failed to induce hypothermia in the 5-HT_1A_ autoreceptor knockout mice. In the FST, Zn potentiated the effect of 8-OH-DPAT. However, in the FST performed with the 5-HT_1A_ autoreceptor knockout mice, the anti-immobility effect of Zn was partially blocked. Both the binding and behavioural studies suggest a concentration-dependent dual mechanism of Zn action at 5-HT_1A_Rs, with potentiation at low dose and inhibition at high dose. Moreover, the in vivo studies indicate that Zn can modulate both presynaptic and postsynaptic 5-HT_1A_Rs; however, Zn’s effects at presynaptic receptors seem to be more potent.

## Introduction

Zinc (Zn) is an essential trace element that is required for proper brain function [1]. Zn modulates neuronal excitability, plays an important role in synaptic plasticity and can function as a signalling molecule [2, 3]. Recent data have indicated that disturbances in Zn homeostasis are involved in the aetiology of some neurological disorders. Several studies, including both preclinical and clinical studies, showed that Zn deficiency leads to the development of depression [4–6] and that Zn supplementation improves the effectiveness of standard antidepressant treatment [7, 8]. One of the possible mechanisms involved in Zn antidepressant activity is the modulation of the serotonergic system through 5-HT_1A_ and 5-HT_2A_ receptors [9, 10]. Zn was found to enhance the effect of citalopram and fluoxetine in the forced swim test (FST) in mice, and pretreatment with an inhibitor of serotonin synthesis, p-chlorophenylalanine (pCPA), blocked the antidepressant-like effect observed in the FST [10]. Moreover, the fact that rats show an increase in the swimming but not the climbing parameter in the FST following Zn administration indicates (according to the observation of Detke et al. [11]) the involvement of the serotonin pathway in the effects of Zn in the FST [10]. Additionally, it was found that the antidepressant-like effect of Zn in the FST in mice was blocked by the 5-HT_1A_R antagonist WAY-100635, which suggested that the modulation of the serotonergic system by Zn is mediated mostly through the 5-HT_1A_R [10].

Further evidence for the postulated direct link between Zn and 5-HT_1A_R were recently provided by Tena-Campos et al. [12] who showed the impact of Zn homeostasis on the balance between monomers/heterodimers of 5-HT_1A_ and galanin 1 receptor (GalR1). The latter protein was indicated as being involved in major depressive disorder by modulating 5-HT_1A_ functionality via specific heterodimerization process [12].

The direct modulatory effect of Zn at the 5-HT_1A_R was also reported by Barrondo and Salles, who described negative allosteric modulatory properties of Zn ions against antagonist ([^3^H]-WAY-100635) and agonist ([^3^H]-8-OH-DPAT) binding at 5-HT_1A_Rs in cortical membranes isolated from the rat brain [13]. It should be noted, however, that the changes observed for [^3^H]-8-OH-DPAT (i.e. a decrease of dissociation constant (*K*_*D*_) and *B*_max_ values were demonstrated through saturation experiments only and were complex and difficult to interpret. Moreover, although studies using native tissue have strong biological significance, membranes prepared from rat cerebral cortex contain other receptors that can be targeted by [^3^H]-8-OH-DPAT (i.e. serotonin 5-HT_7_ receptors and α-2 adrenergic receptors) [14, 15], which might create an additional level of complexity in the interactions of Zn at 5-HT_1A_Rs. As allosteric modulation is strongly probe-dependent [16], evaluation of the effects of Zn on agonist binding is of primary importance to its action observed in vivo. Thus, we performed in vitro radioligand experiments to gain further mechanistic insight into the nature of Zn interactions at 5-HT_1A_Rs. In the present study, HEK293 cells expressing 5-HT_1A_ (5-HT_1A_) receptors were used, which provides a homogeneous system to study effects solely attributable to the 5-HT_1A_R subtype. The influence of Zn ions on agonist binding was investigated by saturation, competition and both association and dissociation kinetic studies using [^3^H]-8-OH-DPAT, a 5-HT_1A_R agonist.

Due their localization, neuronal 5-HT_1A_Rs are divided into two different classes: the 5-HT_1A_ autoreceptors located on the soma and dendrites of serotonergic neurons in the raphe nucleus and the heteroreceptors expressed postsynaptically in the prefrontal cortex (PFC), amygdala and hippocampus [17, 18]. The activation of 5-HT_1A_R has been shown to induce a number of behavioural responses, including lower lip retraction (LLR), flat body posture (FBP), forepaw treading (FT), and a decrease in body temperature [19–23]. The ability of compounds to induce or block these behaviours is commonly used to differentiate and characterize their activity at pre- or postsynaptic 5-HT_1A_Rs. Therefore, in addition to the binding assays, we assessed the ability of Zn to produce LLR, FBP and FT in rats. We also studied both the capacity of Zn to induce hypothermia and the activity of Zn in the FST in rats as well as in wild-type and 5-HT_1A_ autoreceptor knockout mice.

## Methods

### Receptor Binding Studies

#### Drugs

[^3^H]-8-OH-DPAT (135.2 Ci/mmol) was purchased from PerkinElmer and (R)-(+)-8-OH-DPAT, 5-HT and ZnCl_2_ were obtained from Sigma-Aldrich.

#### Expression of the Gene for the Human 5-HT_1A_R

The full-length human 5HTR_1A_ complementary DNA (cDNA), which was cloned into the mammalian expression vector pcDNA3.1(+), was obtained from the Missouri S&T cDNA Resource Center (www.cdna.org). The receptor cDNA was stably transfected into human embryonic kidney cells (HEK293, ATCC) with the use of Lipofectamine 2000 (Invitrogen). A clone yielding a high expression level of 5-HT_1A_R was selected during preliminary experiments, including RT-PCR and Western blot analysis.

#### Cell Culture and Preparation of Cell Membranes

HEK293 cells with stable expression of 5-HT_1A_R were maintained at 37 °C in a humidified atmosphere with 5 % CO_2_ and were grown in Dulbecco’s Modified Eagle Medium (Lonza Ltd.) containing 10 % dialysed foetal bovine serum (Lonza Ltd.) and 500 μg/ml G418 sulphate (Sigma-Aldrich). For membranes preparations, the cells were subcultured in 150 cm^2^ flasks, grown to 90 % confluence, washed twice with phosphate buffered saline (PBS) prewarmed to 37 °C and pelleted by centrifugation (200 *g* for 5 min) in PBS containing 0.1 mM EDTA and 1 mM dithiothreitol. Prior to membrane preparation, the pellets were stored at −80 °C.

#### Preparation of Membranes for Radioligand Binding Assays

Cell pellets were thawed and homogenized in 20 volumes of 50 mM Tris–HCl buffer (pH 7.7) containing 0.1 mM EDTA and 10 mM MgCl_2_, using an Ultra Turrax tissue homogenizer. The pellets were then centrifuged twice at 35,000 *g* for 20 min at 4 °C, with incubation for 15 min at 37 °C in between centrifugations. Membranes were aliquoted in tubes. Membrane protein concentrations were determined using the Pierce™ Coomassie (Bradford) Protein Assay Kit, with bovine serum albumin (BSA) as a standard.

#### Radioligand Binding Assays

[^3^H]-8-OH-DPAT was used as a selective 5-HT_1A_R agonist. The affinity (*K*_D_) and maximal number of binding sites (*B*_max_) were measured by saturation binding experiments over a radioligand concentration range of 0.1–14 nM. The affinity shift was determined by measuring the *K*_D_ obtained in saturation binding assays performed in the absence and presence of six concentrations of Zn (0.01–5 mM). Competition studies were performed with 2.5 nM of [^3^H]-8-OH-DPAT in the presence of various concentrations of orthosteric agonist serotonin in the absence and presence of Zn at two concentrations (10 and 500 μM). Non-specific binding was estimated in the presence of 10 μM 5-HT. The incubation buffer consisted of 50 mM Tris–HCl (pH 7.7), 10 mM MgCl_2_, 10 mM pargyline and 0.1 % ascorbic acid. Radioligand binding assays were performed by incubating 30 μg of protein of the membrane suspension in 96-well microtitre plates for 60 min at room temperature with shaking, in a total volume of 200 μl. The binding reactions were stopped by filtration through GF/C Unifilter plates using a harvester (PerkinElmer). The plate filters were dried, and 20 μl of Ultima Gold MV (PerkinElmer) was added. Radioactivity was measured using a MicroBeta TriLux counter (PerkinElmer).

#### Association and Dissociation Assays

Association and dissociation rate kinetic assays were performed at room temperature using the same buffer conditions described for the equilibrium binding assays and 2.5 nM [^3^H]-8-OH-DPAT. Non-specific binding was defined by the addition of 10 μM serotonin. The amount of radioligand bound to the receptor was measured at different time intervals during a total incubation of 60 min in the absence or presence of 10 and 500 μM ZnCl_2_. For the dissociation assay, after incubating the membranes with radioligand for 60 min to achieve equilibrium, serotonin (10 μM), either alone or together with 10 or 500 μM of ZnCl_2,_ was added, and the specifically bound radioligand was measured after incubations of different durations (from 0 to 60 min), which were terminated by rapid filtration.

#### Data Analysis

All experiments were performed in triplicate, and the results were obtained from at least three independent experiments. The data are expressed as the mean ± S.D. (standard deviation). The experimental data were analysed using GraphPad Prism 5.1 for Windows (GraphPad Software, San Diego California USA, www.graphpad.com). Analysis of the saturation binding data with respect to allosteric interactions was performed with the use of equation (1) [24]:

$$ p{K}_{\mathrm{App}}=- \log \left(\left[B\right]+{10}^{\log {K}_B}\right)+ \log \left(\alpha \left[B\right]+{10}^{\log {K}_B}\right)- \log d $$

where *K*_App_ is the apparent equilibrium dissociation constant of radioligand *A* ([^3^H]-8-OH-DPAT) observed in the presence of modulator B (ZnCl_2_); *K*_*A*_ and *K*_*B*_ are the equilibrium dissociation constants of the radioligand and allosteric modulator, respectively; log*d* is a constant representing the logarithm of the quotient of *K*_*A*_ and *α*; and *α* defines the cooperativity factor, the magnitude by which the equilibrium dissociation constant of either ligand for its site on the receptor is modified by the concomitant presence of the other ligand. Values of *α* less than 1 (but greater than zero) denote negative cooperativity, values greater than 1 denote positive cooperativity and values not significantly different from 1 indicate neutral cooperativity.

Bell-shaped concentration-response curves were fit to a special model [16], based on equation (2):

$$ {\rho}_A=\frac{\frac{\left[A\right]}{K_A}\left(1+\frac{\alpha^{\prime}\left[B\right]}{K_{B2}}\right)}{\frac{\left[A\right]}{K_A}\left(1+\frac{\alpha^{\prime}\left[B\right]}{K_{B2}}\right)+1+\frac{\left[B\right]}{K_{B1}}+\frac{\left[B\right]}{K_{B2}}\left(1+\frac{\beta \left[B\right]}{K_{B1}}\right)} $$

where ρ_A_ denotes the fractional receptor occupancy by the orthosteric ligand; [*A*] and [*B*] are the concentrations of [^3^H]-8-OH-DPAT and ZnCl_2_, respectively; *K*_*A*_, *K*_*B1*_, and *K*_*B2*_ denote equilibrium dissociation constants; the subscript ‘1’ refers to the binding of B to the orthosteric site; the subscript ‘2’ refers to the binding of B to the allosteric site; the cooperativity factor *α′* denotes the interaction between *B* and *A*; and *β* denotes the interaction between the two molecules of *B*.

The experimental data were fit to one-site and two-site models to determine the best fit. The statistical significance of differences between the means was evaluated by Student’s *t* test. The level of significance was set at *p* < 0.05.

### Behavioural Studies

#### Animals and Housing

The experiments were performed on male Albino Swiss mice (23–25 g) and Sprague Dawley rats (200–250 g) from Charles River, Germany as well as TPH2-Cre-ER^T2^ x flx-5-HT_1A_-flx-YFP mice from Jackson Labs, Bangor ME and the Hen lab, respectively. The animals were kept under standard laboratory conditions with respect to lighting (light phase 7:00–19:00) and temperature (19–21 °C). Food and water were freely available. Each experimental group consisted of eight to ten animals. All of the animals were experimentally naive and were used only once in each test. The experiments were performed during the light period (9:00–14:00 h). All of the procedures were conducted according to the guidelines of the National Institutes of Health Animal Care and Use Committee and were approved by the Ethics Committees of the Institute of Pharmacology, Polish Academy of Sciences in Krakow and the Animal Care Committee, University of Ottawa.

#### Drug Administration

Zn (all doses refer to mg Zn/kg) was given as either Zn hydroaspartate (Farmapol, Poland) or Zn chloride (Sigma-Aldrich) and was dissolved in 0.9 % NaCl and administered intraperitoneally (*i.p.*). 8-OH-DPAT and WAY-100635 were dissolved in aqua pro-injection and were administered subcutaneously (*s.c.*). Controls were treated with the appropriate diluent, which was indicated as VEH on the graphs and in the tables.

#### Presynaptic 5-HT_1A_R Knockout Mice

A conditional knockout approach was used to eliminate the 5-HT_1A_R in the raphe nuclei in adult mice. To create the conditional 5-HT_1A_R knockout mice, a TPH2-Cre-ER^T2^ mouse line was crossed with a flx-5-HT_1A_-YFP mouse line. The TPH2-Cre-ER^T2^ confers specificity such that only serotonin neurons, which express tryptophan hydroxylase 2 (TPH2), can express Cre recombinase to knockout the 5-HT_1A_R gene and allow for GFP expression [25]. Once the mice reached adulthood (approximately 5–6 postnatal weeks), tamoxifen (180 mg/kg) was injected *i.p.* every day for five consecutive days (the tamoxifen binds to the ER^T2^ domain and enables Cre to enter the nucleus where it can excise the 5-HT_1A_R gene, allowing for YFP expression as a marker of recombination). The mice were then left undisturbed for at least 14 days to allow for recombination and the turnover of endogenous 5-HT_1A_Rs.

#### Lower Lip Retraction

LLR was assessed according to the method described by Berendsen et al., [20]. The rats were individually placed in cages (30 × 25 × 25 cm) and were scored three times, 15, 30 and 45 min after the administration of Zn as follows: 0 = lower incisors not visible, 0.5 = partly visible and 1 = completely visible. Zn was administered at doses of 2 and 5 mg Zn/kg (given as Zn hydroaspartate, Farmapol, Poland). The maximum total score was 3/rat. In addition, the effects of Zn and WAY-100635 (0.1 mg/kg; Sigma) on the LLR induced by 8-OH-DPAT (1 mg/kg; Sigma) were tested. Zn and WAY-100635 were administered 45 min before 8-OH-DPAT, and the animals were scored 15, 30 and 45 min after the 8-OH-DPAT administration.

#### Behavioural Syndrome

The experiments were conducted according to previously published procedure [26], with a modification to the ranked intensity scale. Briefly, the rats were individually placed in cages (30 × 25 × 25 cm) 5 min before tested compounds were injected. Forepaw treading (FT) and flat body posture (FBP) were scored using a ranked intensity scale, where 0 = absent, 1 = equivocal and 2 = present. The Zn (2, 5, 7.5 and 11.5 mg Zn/kg, given as a Zn hydroaspartate)-induced behavioural syndrome was scored for each animal 3, 6, 9, 12 and 15 min after the Zn treatment. Each observation session lasted for 45 s. The maximum score, which was summed over 5 observation periods, was 10 for each symptom/rat. The effect of Zn on the behavioural syndrome induced by 8-OH-DPAT (5 mg/kg) was scored using the same scale. Zn was administered 60 min before 8-OH-DPAT, and the animals were scored 3, 6, 9, 12 and 15 min after the 8-OH-DPAT treatment.

#### Body Temperature in Mice and Rats

The effects of Zn (2, 5, 7.5 and 11.5 mg Zn/kg for rats and 2 and 5 mg/kg for mice, given as a Zn hydroaspartate), 8-OH-DPAT (5 mg/kg) and WAY-100635 (0.1 mg/kg) on rectal body temperature were recorded 30 and 60 min after their acute administration. The results were expressed as a change in body temperature with respect to the basal body temperature, which was measured at the beginning of the experiments.

#### Body Temperature in 5-HT_1A_ Autoreceptor KO Mice

A separate experiment was performed to measure the effect of Zn on rectal body temperature in wild-type and 5-HT_1A_ autoreceptor KO mice; however, in this experiment, only the higher dose of Zn (5 mg Zn/kg; Zn chloride, Sigma) was used.

#### Forced Swim Test (FST)

##### Rats

The test was carried out according to the method described previously [10]. On day one of the experiment, the animals were individually placed in plexiglass cylinders (40 cm in height, 18 cm in diameter), containing 25 cm of water maintained at 24–25 °C for a 15-min habituation period. After the rats were removed from the water, they were again placed in their home cages. On the second day, the rats were again placed in the cylinders, and the total duration of immobility was measured for a 5-min test period. Zn (1 or 2 mg Zn/kg, given as Zn hydroaspartate) and 8-OH-DPAT (0.1 or 0.3 mg/kg) were administered alone or jointly 30 min before the test. WAY-100635 (0.1 mg/kg) was administered 15 min before the Zn treatment.

##### 5-HT_1A_ Autoreceptor KO Mice

The behaviour of the mice in the water was recorded using a video camera. The test was performed under red lighting. The cylinder was filled with almost 4 l of water, to a depth that exceeded the distance to which the tail could extend, so the mouse could not balance on its tail at the bottom of the cylinder. The top of the cylinder was 9 cm above the surface of the water. The mice were placed in the individual glass cylinder (22 cm diameter × 37 cm high) for a standard 6-min test; however, the total duration of immobility from the last 4 min of the test was analysed. In this test, only one dose of Zn—5 mg Zn/kg (given as a Zn chloride)—was administered 30 min before the test.

### Data Analysis

One-way ANOVA followed by Dunnett’s multiple comparison test (LLR, FBP, FT and FST) or Student’s *t* test (FST and body temperature in wild-type and 5-HT_1A_ autoreceptor KO mice) was used, and *p* < 0.05 was considered significant.

## Results

### Receptor Binding Studies

**Fig. 1:**
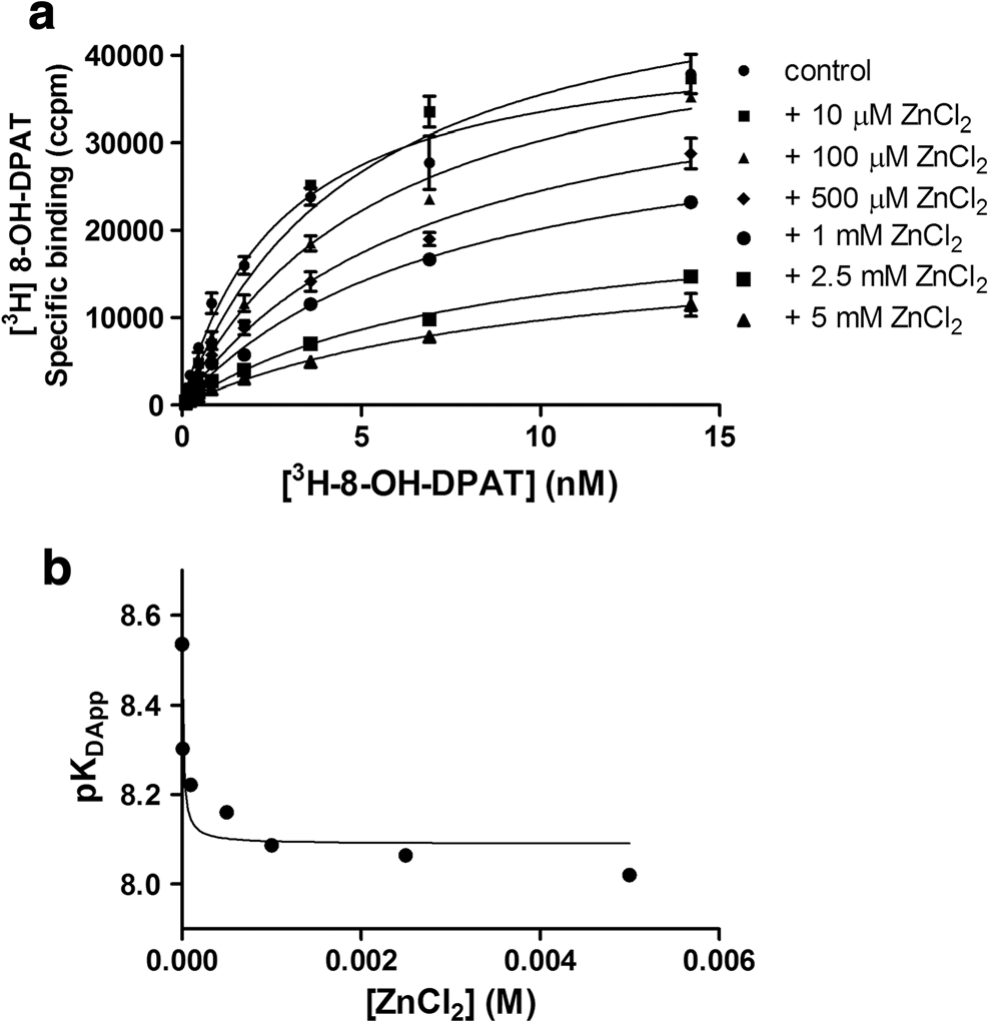
Effect of increasing concentrations of Zn on the saturation binding of [^3^H]-8-OH-DPAT in 5-HT_1A_ receptor-expressing HEK293 cells. **a** Representative set of radioligand saturation binding curves obtained in the absence and presence of Zn. **b** Nonlinear regression analysis of the saturation data according to equation 1

The binding of [^3^H]-8-OH-DPAT to 5-HT_1A_Rs was saturable, yielding an equilibrium constant of *K*_*D*_ = 2.9 ± 0.1 nM (*n* = 4) and a maximal receptor binding of *B*_max_ = 4.5 ± 0.3 pM/mg prot. Saturation isotherms obtained for six increasing concentrations of Zn (10 μM–5 mM) revealed a decrease in radioligand binding (Fig. 1a). The *K*_*D*_ values for [^3^H]-8-OH-DPAT increased from 5 nM at 10 μM of Zn to 9.6 nM at 5 mM of Zn relative to the *K*_*D*_ obtained for [^3^H]-8-OH-DPAT without Zn (Table 1). At the same time, unusual changes in *B*_max_ values were observed. Zn at 10 μM caused an increase in specific binding (*B*_max_ = 5.8 ± 0.7 pM/mg prot.), Zn at 0.5 and 1 mM recovered the control values (4.7 ± 0.7 and 4.0 ± 0.6 pM/mg prot., respectively), and the two highest concentrations of Zn (2.5 and 5 mM) significantly decreased *B*_max_ values (3.1 ± 1.0 and 2.2 ± 0.8 pM/mg prot., respectively).

**Table 1:** Effects of Zn^2+^ on *K*_*D*_ and *B*_max_ values of [^3^H]8-OH-DPAT obtained in saturation binding experiments in 5-HT_1A_ receptors in HEK293 cells

Zn^2+^ [μM]	0	10	100	500	1000	2500	5000
*K*_*D*_ [nM]	2.9 ± 0.1	5.0 ± 0.1	6.0 ± 0.4	6.9 ± 0.2	8.2 ± 0.9	8.6 ± 0.6	9.6 ± 0.6
*B*_max_ [pM/mg prot]	4.5 **±** 0.3	5.8 **±** 0.7*	6.2 **±** 2.0	4.7 **±** 0.7	4.0 **±** 0.6	3.1 **±** 1.2*	2.5 **±** 0.8*

*p < 0.05

The data derived from the saturation experiments were fit using nonlinear regression according to equation 1 (Fig. 1b), and the calculated value of the cooperativity factor (*α* = 0.37) indicated negative modulation between the binding of Zn and the agonist radioligand. This is generally consistent with the results described by Barrondo and Salles for rat cortical membranes, except that increased *B*_max_ values at lower Zn concentrations were not observed in their saturation studies [13].

**Fig. 2:**
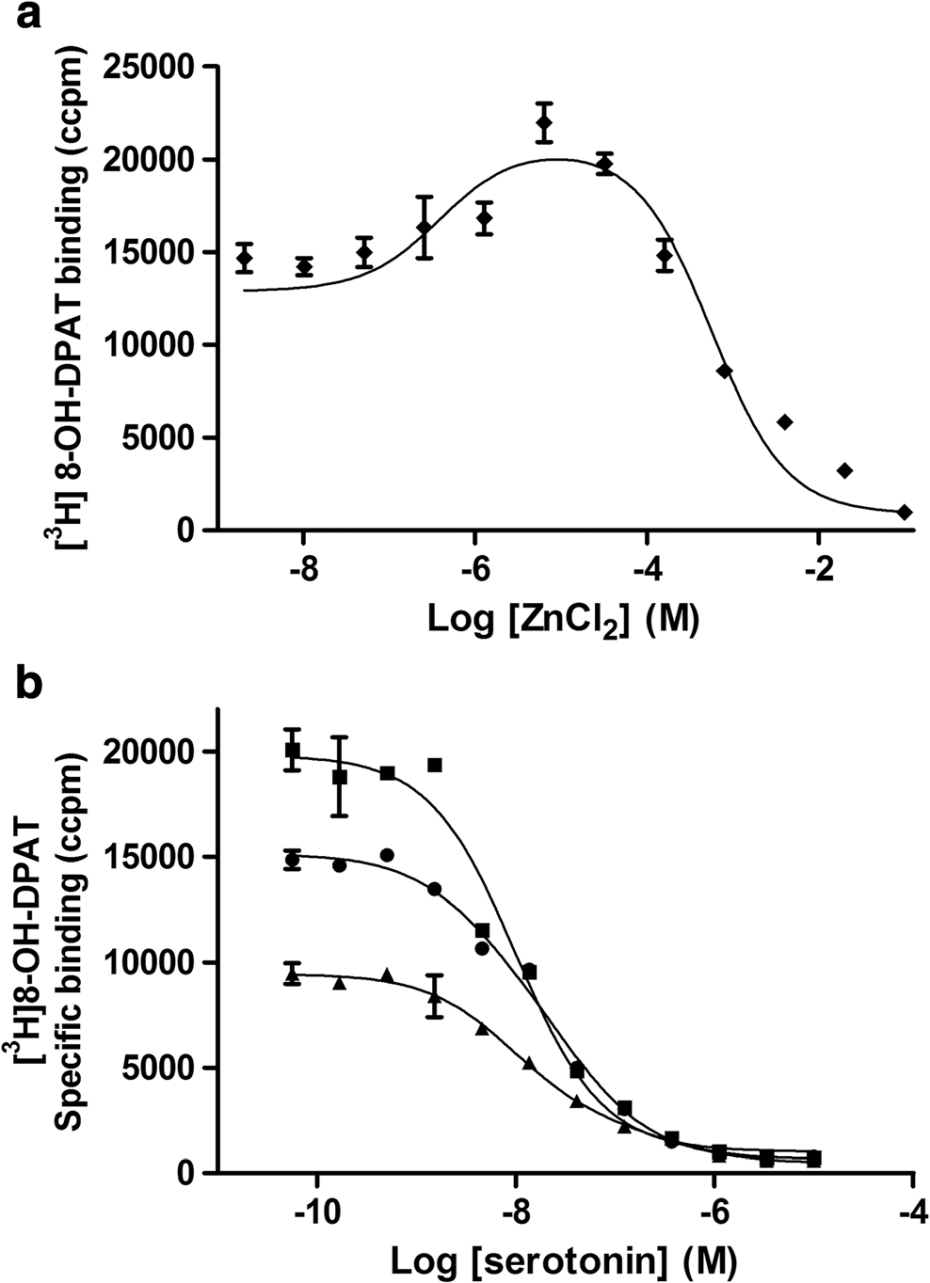
The ‘bell-shaped’ Zn titration curve obtained in the competition-like experiments, with the enhancement (~10 μM of Zn) and inhibition (above 100 μM of Zn) of [^3^H]-8-OH-DPAT binding at 5-HT_1A_Rs. The curve was generated by fitting the data to equation 2 (**a**). Displacement of specific [^3^H]-8-OH-DPAT binding by 5-HT in the absence (*black circle*; *K*_*i*_ = 8.7 ± 0.6 nM) or presence of 10 μM (*black square*; *K*_*i*_ = 5.4 ± 0.2 nM; *p* < 0.05) and 500 μM (*black triangle*; *K*_*i*_ = 8.1 ± 0.6 nM) of Zn (**b**)

Next, a Zn titration curve against a single, fixed concentration of [^3^H]-OH-DPAT (2.5 nM) was evaluated in competition-like experiments. As observed in Fig. 2a, a bell-shaped binding curve was obtained, with an ~25 % increase of [^3^H]-8-OH-DPAT specific binding at 10 μM of Zn and subsequent inhibition at >100 μM. This type of binding is characteristic of ligands exhibiting some degree of allosteric enhancement; therefore, the classic model of negative cooperativity is not a complete description of the action of Zn ions on agonist binding at 5-HT_1A_Rs. Thus, it was interesting to check the influence of Zn ions on serotonin (as an endogenous orthosteric agonist) in displacement experiments in the absence and presence of 10 and 500 μM of Zn. The 5-HT tested alone completely inhibited [^3^H]-8-OH-DPAT binding at 5-HT_1A_Rs, with a *K*_*i*_ of 8.7 ± 0.6 nM; addition of 10 μM of Zn ions caused a small but significant reduction of K_i_ value for 5-HT (*K*_*i*_ = 5.4 ± 0.2 nM, *p* < 0.05, *F* test), while at higher Zn concentrations the affinity of 5-HT remained unchanged (*K*_*i*_ = 8.1 ± 0.6 nM). Figure 2b shows a representative set of inhibition curves of [^3^H]8-OH-DPAT for 5-HT obtained in the absence and presence of Zn ions.

### Kinetic Studies

It is well known that allosteric modulators may increase or decrease the association and/or dissociation rates of an orthosteric ligand at its binding site in a way that enhancers increase the association rate and/or decrease the dissociation rate, whereas negative allosteric modulators act in the opposite way. On the other hand, competitive orthosteric ligands can influence the association rate by increasing the time needed for the radioligand to reach equilibrium, but do not change the dissociation rate [27].

**Fig. 3:**
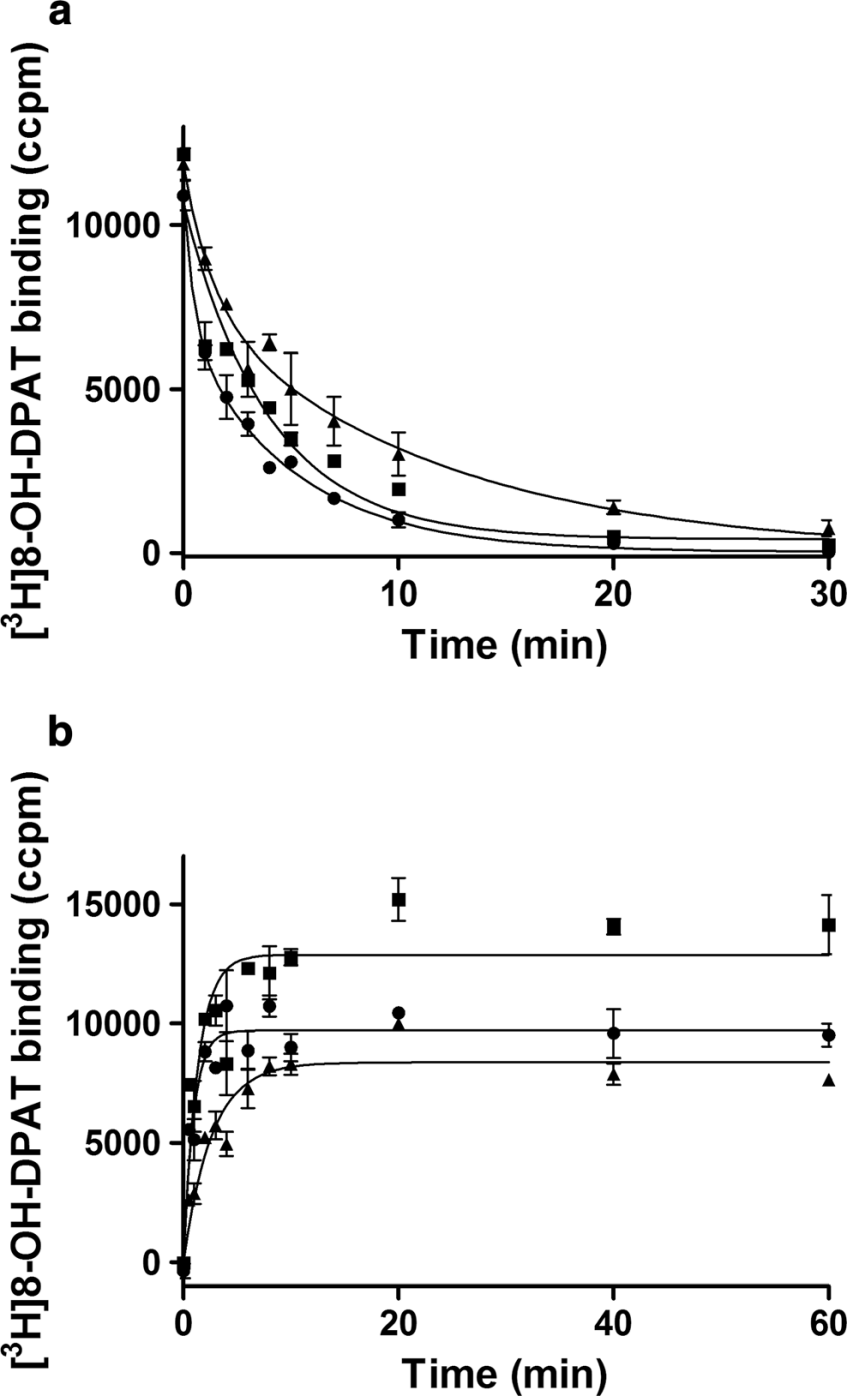
Effect of Zn on the dissociation (**a**) and association (**b**) rates of [^3^H]-8-OH-DPAT in the absence (*black circle*) or presence of 10 μM (*black square*) and 500 μM (*black triangle*) of Zn

Taking into account the complex mechanism of Zn influence on agonist binding, dissociation and association (see Fig. 3) rates were measured in kinetic assays; the values of kinetic parameters are listed in Table 2.

**Table 2:** Association and dissociation rate constants of [^3^H]-8-OH-DPAT obtained in the kinetic experiments in the absence and presence of Zn

	*k*_on_ [M^−1^ min^−1^]	*k*_off fast_ [min^−1^]	*k*_off slow_ [min^−1^]
Control	3.4 ± 0.4 × 10^+8^	2.33 ± 0.31	0.21 ± 0.02
+10 μM Zn^2+^	6.4 ± 0.8 × 10^+8^*	0.25 ± 0.04^a^	
+500 μM Zn^2+^	3.6 ± 0.3 × 10^+8^	0.58 ± 0.07**	0.06 ± 0.02**

*p < 0.07; **p < 0.05

^a^one phase

In the absence of Zn, the dissociation rate for [^3^H]-8-OH-DPAT at 5-HT_1A_Rs was biphasic, and it remained biphasic at high Zn concentration, but both phases were significantly reduced without affecting the proportion of each state. However, the presence of a low concentration of Zn led to the disappearance of the fast radioligand dissociation rate, and the kinetics of [^3^H]-8-OH-DPAT became monophasic. These results suggest that Zn modifies receptor conformation, reducing agonist radioligand dissociation, which is in line with the enhancing effects exerted by Zn in the competition-like experiments.

As detailed in Table 2, [^3^H]-8-OH-DPAT (2.5 nM) binds at 5-HT_1A_Rs with an association rate constant (k_on_) of 3.4 ± 0.4 × 10^8^ M^−1^ min^−1^. In the presence of 10 μM of Zn ions, a small increase in the association rate was observed, while at a higher concentration (500 μM), Zn had no effect on the k_on_ values.

### Behavioural Studies

#### Effects of Zn Treatment on the LLR, FBP and FT in Rats

8-OH-DPAT (1 mg/kg), the 5-HT_1A_ agonist, induced LLR (*p* < 0.001) in the rats. Zn, given at a dose of 2 and 5 mg/kg, and WAY-100635, a 5-HT_1A_ antagonist, given at the dose of 0.1 mg/kg, did not evoke LLR; however, they blocked the LLR induced by 8-OH-DPAT (Zn: *p* < 0.05 for the dose of 2 mg/kg and *p* < 0.001 for the dose of 5 mg/kg; *p* < 0.01 for WAY-100635, vs. 8-OH-DPAT) (see Table 3). When given at the doses of 2, 5, 7.5 and 11.5 mg/kg, Zn did not induce FBP or FT in the rats (see Table 4); however, at the higher doses of 11.5 mg/kg (*p* < 0.001), 7.5 and 11.5 mg/kg (*p* < 0.001 and *p* < 0.01, respectively), Zn blocked the 8-OH-DPAT-induced FBP and FT.

**Table 3:** Lower lip retraction score as a function of 8-OH-DPAT for the Zn, WAY-100635 and control groups

	Mean ± LLR score	
Treatment (mg/kg)	(A) VEH	(B) 8-OH-DPAT 1 mg/kg
VEH	0.0 ± 0.0	2.25 ± 0.46^###^
Zn 2	0.0 ± 0.0	1.17 ± 0.98*
Zn 5	0.0 ± 0.0	0.46 ± 0.75***
WAY 100635 0.1	0.0 ± 0.0	0.67 ± 0.76**

(A) Each animal was scored for LLR three times, 15, 30 and 45 min after the administration of Zn as follows: 0 = lower incisors not visible, 0.5 = partly visible and 1 = completely visible. The maximum total score was 3/rat. Zn was administered at doses of 2 and 5 mg Zn/kg. (B) The effect of Zn and WAY-100635 on the LLR induced by 8-OH-DPAT was scored using the same scale. Zn and WAY-100635 was administered 45 min before 8-OH-DPAT and the animals were scored 15, 30 and 45 min after 8-OH-DPAT treatment. The values represent the mean ± SEM (*n* = 8–10 rats per group)

^###^ *p* < 0.01 vs. VEH; *p < 0.05, **p < 0.01 and ***p < 0.001 vs. 8-OH-DPAT. Statistical analysis was performed using one-way ANOVA followed by Dunnett’s post hoc test

**Table 4:** Induction of behavioural syndrome by Zn (A) and the effect of Zn on the 8-OH-DPAT-induced behavioural syndrome (B)

Treatment	Dose (mg/kg)	Mean ± SEM behavioural score			
		(A) VEH		(B) 8-OH-DPAT	
		FBP	FT	FBP	FT
VEH		0.0 ± 0.0	0.0 ± 0.0	7.83 ± 0.7	7.5 ± 0.6
Zn	2	0.2 ± 0.2	0.3 ± 0.2	7.7 ± 0.3	7.1 ± 0.6
	5	0.3 ± 0.3	0.2 ± 0.2	7.5 ± 0.8	7.5 ± 0.5
	7.5	0.5 ± 0.5	0.2 ± 0.2	7.0 ± 0.3	5.0 ± 0.6**
	11.5	0.5 ± 0.2	0.2 ± 0.2	3.4 ± 0.6***	0.4 ± 0.2***

(A) In each animal, the Zn-induced behavioural syndrome was scored at 3, 6, 9, 12 and 15 min after Zn treatment. FBP, flat body posture, and FT, forepaw treading, were scored using the following scale: 0 = absent, 1 = equivocal, and 2 = present. The maximum score, which was summed over 5 observation periods, was 10 for each symptom/rat. (B) The effect of Zn on the behavioural syndrome induced by 8-OH-DPAT was scored using the same scale. Zn was administered 60 min before 8-OH-DPAT (5 mg/kg), and the animals were scored at 3, 6, 9, 12 and 15 min after 8-OH-DPAT treatment. The data represent the mean ± SEM for *n* = 6 rats

***p < 0.001; **p < 0.01, compared to the VEH + 8-OH-DPAT group. Statistical analysis was performed using one-way ANOVA followed by Dunnett’s post hoc test

#### Effects of Zn Treatment on the Body Temperature of Rats and Mice

**Fig. 4:**
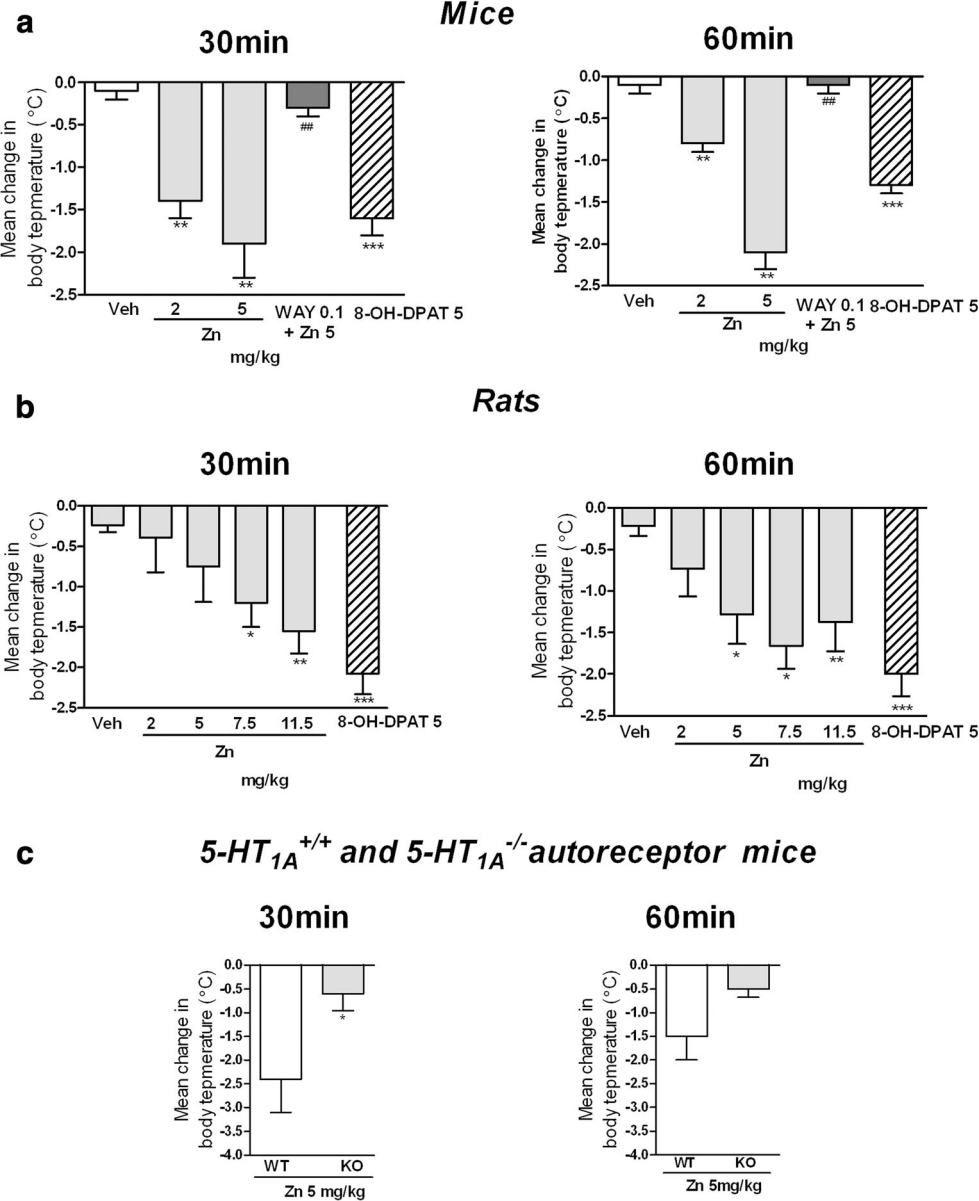
Effect of Zn and 8-OH-DPAT on body temperature in mice (**a**), rats (**b**) and 5-HT_1A_^+/+^ (WT) and 5-HT_1A_ autoreceptor^−/−^ (KO) mice (**c**). The change in body temperature was calculated for each animal by comparing the baseline temperature to the temperature reached 30 and 60 min following Zn or 8-OH-DPAT administration. WAY-100635 at a dose of 0.1 mg/kg was administered *(sc)* 15 min before the Zn (**a**). The test was performed 30 and 60 min after injection of Zn (5 mg/kg) *(ip)*. The *data bars* represent the mean ± SEM for *n* = 8 (mice); *n* = 5 (WT mice) and *n* = 8 (KO mice) and *n* = 6 (rats) animals. *p < 0.05; **p < 0.01; ***p < 0.001, compared to the VEH group. Statistical analysis was performed using one-way ANOVA followed by Dunnett’s post hoc test (**a** and **b**) and Student’s *t* test (**c**)

As shown in Fig. 4, 8-OH-DPAT (5 mg/kg) induced a significant decrease in body temperature of the mice (Fig. 4a) and in rats (Fig. 4b) compared to saline group (*p* < 0.001). Similarly to 8-OH-DPAT, Zn significantly and dose-dependently decreased body temperature of the mice at both 30 (*p* < 0.01) and 60 min (*p* < 0.01) after administration (Fig. 4a). Zn also significantly decreased the body temperature of the rats, at both 30 min (*p* < 0.01 for the dose of 11.5 mg/kg, and *p* < 0.05 for the dose of 7.5 mg/kg) and 60 min after the injection (*p* < 0.01 for the dose of 11.5 mg/kg; *p* < 0.01 for the dose of 7.5 mg/kg; and *p* < 0.05 for the dose of 5 mg/kg) (Fig. 4b).

#### Effects of Zn Treatment on the Body Temperature of the 5-HT_1A_ Autoreceptor KO Mice

The effect of Zn treatment on the body temperature of the 5-HT_1A_ autoreceptor KO mice was shown in Fig. 4c. There was no difference in basal body temperature between the wild-type and 5-HT_1A_ autoreceptor KO mice (37.7 ± 0.23 and 37.9 ± 0.19, respectively). Zn administered at a dose of 5 mg /kg, decreased the body temperature of the wild-type mice but not of the autoreceptor KO mice (*p* < 0.05 at 30 min and *p* = 0.06 at 60 min).

#### Effect of Zn in the FST

##### Rats

**Fig. 5:**
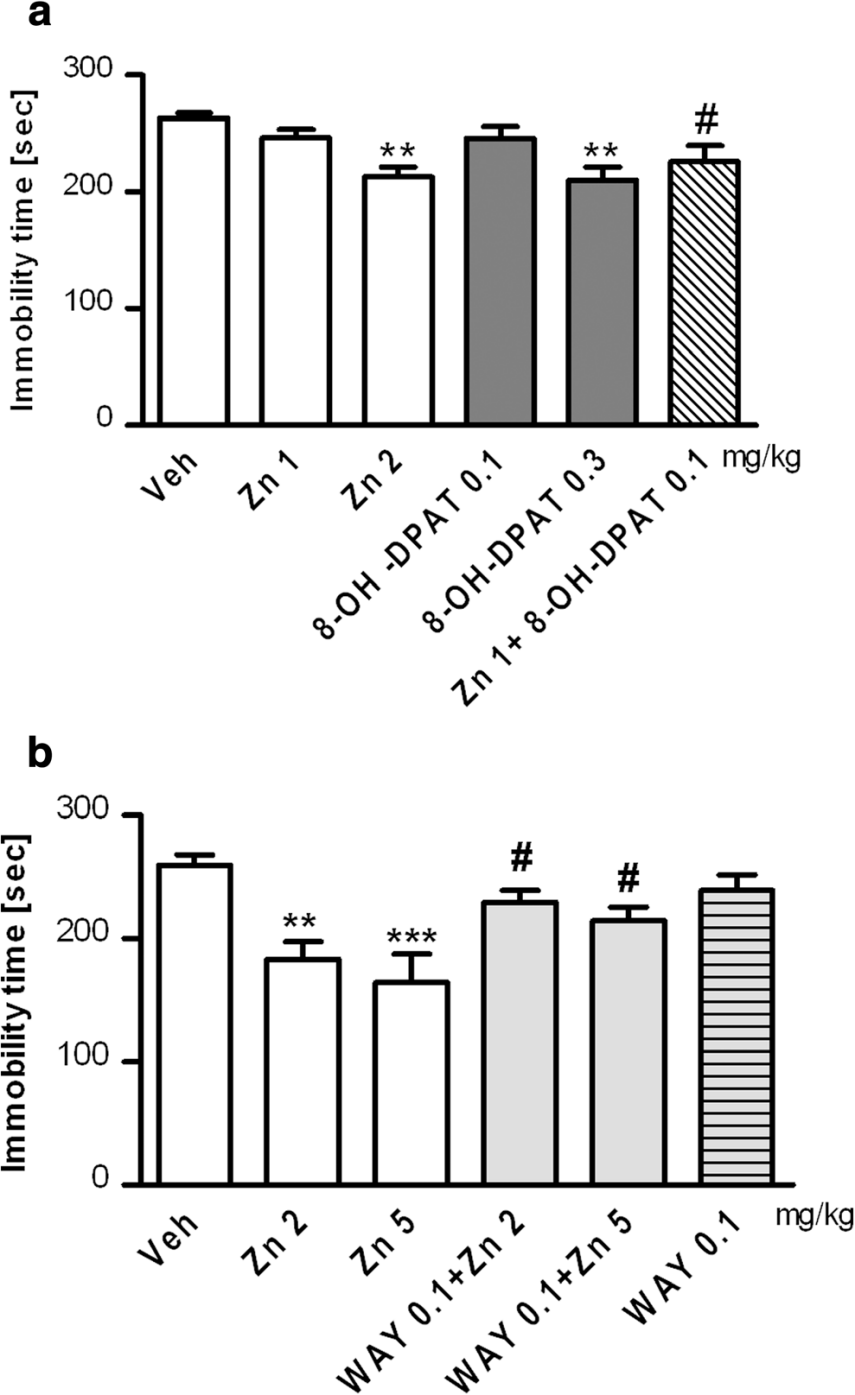
Effect of Zn and 8-OH-DPAT given alone and jointly using the doses that did not affect immobility time in the FST in rats (**a**) and the effect of pretreatment with WAY-100635 on Zn-induced reduction in immobility time (**b**). Zn and 8-OH-DPAT were administered alone or jointly 30 min before the FST (**a**). WAY-100635 was administered 15 min before the Zn treatment, and the FST test was performed 30 min after Zn treatment (**b**). The values represent the mean ± SEM (*n* = 5–8 rats). *p < 0.05; **p < 0.01 vs. the VEH group; ^#^ *p* < 0.05 vs. the Zn treatment group. Statistical analysis was performed using one-way ANOVA followed by Dunnett’s post hoc test

As shown in Fig. 5a, Zn administered at a dose of 2 mg/kg but not 1 mg/kg significantly decreased the immobility time of the rats in the FST (*p* < 0.01 and *p* > 0.05, respectively). 8-OH-DPAT administered at a dose of 0.3 mg/kg (*p* < 0.05) but not 0.1 mg/kg (*p* > 0.05) decreased the immobility time of the rats in the FST. Zn given jointly with 8-OH-DPAT at the doses that were ineffective in the FST significantly decreased the immobility time of the rats (*p* < 0.01) compared to the VEH-treated group. As shown in Fig. 5b, Zn at a dose of 2 and 5 mg/kg significantly decreased the immobility time of the rats in the FST (*p* < 0.05 and *p* < 0.01, respectively). When given alone, WAY-100635, an antagonist of 5-HT_1A_Rs (0.1 mg/kg), did not change the behaviour of the animals in this test, but it antagonized the Zn-induced decrease in the immobility time of the rats (*p* < 0.05 vs. the Zn-treated groups).

##### 5-HT_1A_ Autoreceptor KO Mice

**Fig. 6:**
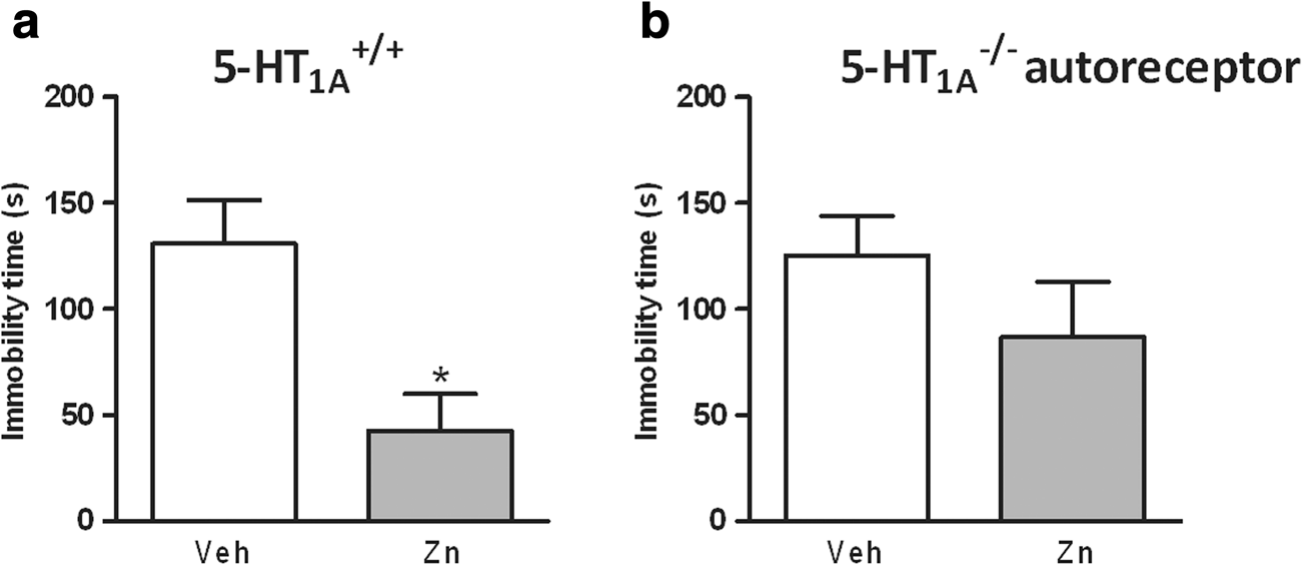
The effect of Zn treatment on the immobility time in the FST in the 5-HT_1A_^+/+^ (WT) (**a**) and 5-HT_1A_ autoreceptor^−/−^ (KO) (**b**) mice. Zn at the dose of 5 mg/kg was administered 30 min before the FST. The *data bars* represent the mean ± SEM for *n* = 3–5 mice. *p < 0.05 compared to the VEH group. Statistical analysis was performed using Student’s *t* test

Zn administered at a dose of 5 mg/kg induced a slight (30 %) decrease in the immobility time of the 5-HT_1A_ autoreceptor KO mice compared to the VEH-treated KO mice (see Fig. 6b), while in wild-type mice (see Fig. 6a), a significant decrease in immobility time after Zn treatment was observed (*p* < 0.05).

## Discussion

### Receptor Binding Studies

As an endogenous trace element, Zn has been suggested to act as an allosteric modulator of a number of G protein-coupled receptors, such as dopamine D_1_, D_2_ and D_4_; melanocortin MC_1_ and MC_4_; α_1A_ and β_2_ adrenergic; μ, κ and δ opioid; and serotonin 5-HT_1A_ receptors [28]. Despite the previous identification of Zn ions as allosteric inhibitors of both agonists and antagonists of 5-HT_1A_Rs [13], the exact mechanism of Zn’s action in relation to orthosteric agonists appears complex and is not fully understood. Thus, in the present study, an extended set of in vitro radioligand binding experiments (saturation, competition-like and kinetic tests) were undertaken using [^3^H]-8-OH-DPAT as an agonist probe to further characterize the effects of Zn on 5-HT_1A_Rs.

Consistent with the data presented by Barrondo and Salles for native tissue [13], the results of the current saturation experiments using stable expression of 5-HT_1A_Rs in HEK293 cells suggest negative allosteric modulation of Zn on [^3^H]-8-OH-DPAT binding (*α* = 0.37). Nevertheless, a model of negatively cooperative interactions does not account for all of the additional data obtained in the study.

At first, Zn tested alone in a competition-like assay with [^3^H]-8-OH-DPAT yielded a bell-shaped binding curve, with a marked increase in agonist radioligand binding at low modulator concentrations and a decrease in binding at high concentrations (Fig. 2a). This type of curve was also shown for several adenosine *A*_1_ allosteric modulators investigated in agonist radioligand binding assays [27, 29, 30]. The results of those studies have invariably been interpreted as evidence that the investigated molecules recognize the allosteric site at low concentrations but also bind to the orthosteric site at higher concentrations, combining the two mechanisms of action: allosteric enhancement and competitive inhibition [27, 29, 30].

Interestingly, in the competition experiments, an enhancement of agonist binding to 5-HT_1A_Rs was evident in the presence of 10 μM of Zn for 5-HT only, while a concentration of 500 μM of Zn did not change the *K*_*i*_ values for this orthosteric agonist. Likewise, the association kinetics of [^3^H]-8-OH-DPAT showed small increase in association rate (*k*_on_) at a low (10 μM) concentration of Zn ions (Table 2), and the lack of statistically significant effects on *k*_on_ values at a higher Zn concentration (500 μM) was observed (Table 2, Fig. 3). However, the results of the dissociation kinetic experiments showed that both concentrations of Zn used slowed the radioligand dissociation rates, which is a characteristic of positive allosteric modulation (Table 2, Fig. 3).

The complex pattern of presently obtained data, when the behaviour of Zn in the saturation experiments and competition and kinetic assays are compared, supports the conclusion that the previously assumed negatively cooperative model is not a complete description of Zn’s effects on agonist binding. Because Zn displayed negative cooperative interactions with [^3^H]-8-OH-DPAT in the saturation experiments but caused an increase in *B*_max_ values at low concentrations and directly showed properties of positive allosteric modulation in both the competition-like and kinetic experiments, a dual mode of Zn action against agonist binding at 5-HT_1A_Rs should be considered.

It is worth noting that in the case mentioned above, in which adenosine *A*_1_ ligands showed positive cooperativity in interactions with agonists, when the same modulators were tested against antagonist probes, they were characterized only by inhibitory properties [29, 30]. Similar probe dependence can be observed by comparing the data we obtained for the effects of Zn on agonist binding at 5-HT_1A_Rs with earlier results describing clear negative modulation of antagonist ([^3^H]-WAY-100635) binding [13].

It should be mentioned that a complex mechanism of Zn action has also been detected for several other proteins. In electrophysiological studies of 5-HT_3_ receptor, low concentrations of Zn (0.3–10 μM) enhanced and high concentrations of Zn (30–200 μM) depressed, the 5-HT-induced response [31]. Biphasic effects involving potentiation at sub-micromolar and inhibition at sub-millimolar Zn concentrations have been detected for glycine receptors [32]. Similar effects were also demonstrated for *β*_2_ adrenergic receptors, in which the presence of 5 μM of Zn enhanced agonist affinity, whereas 500 μM of Zn inhibited antagonist binding [33].

Summing up, Zn released into synaptic space from neuronal vesicles (of mostly glutamatergic terminals) was found to act at various channels and membrane receptors and these modulatory effects of Zn can be positive or negative depending on its concentration. The exact amount of Zn release is controversial; however, many laboratories have indicated that Zn increases in the extracellular space may reach 1–100 μM [34–36]. Therefore, it seems that the effects observed at lower Zn concentrations should be physiologically more relevant, especially that the elevation of extracellular Zn level, over 300 μM, is reported as neurotoxic [37].

### In Vivo Studies

The results of in vivo studies provide evidence that Zn is likely to have both an agonist and antagonist profile at 5-HT_1A_ receptors that could be a consequence of dual Zn effects at 5-HT_1A_ receptors suggested by in vitro studies.

### Body Temperature

Induction of hypothermia in response to the administration of 8-OH-DPAT is one of the parameters which have been proposed as an index of 5-HT_1A_R autoreceptor mediated activity [23, 38]. In our study, Zn decreased the body temperature in mice and the intensity of this effect was similar to that observed for 8-OH-DPAT. Furthermore, pretreatment with WAY-100635, the 5-HT_1A_R antagonist, abolished the effect induced by Zn. On the other hand, our studies using 5-HT_1A_ autoreceptor KO mice showed that lack of this receptor completely blocked the hypothermia induced by Zn, while in wild-type littermate mice, a consistent decrease in body temperature was observed. These results are consistent with those showing that hypothermic effect of 5-HT_1A_R agonists in mice is mediated by presynaptic 5-HT_1A_R [38, 39] and indicate an agonist-like profile of Zn at presynaptic 5-HT_1A_Rs, perhaps enhancing the action of endogenous 5-HT.

In the present studies, we found that Zn can also induce hypothermia in rats; however, this effect was observed at higher doses than in mice. It is still controversial which 5-HT_1A_Rs namely pre- or postsynaptic are responsible for the induction of decrease in the body temperature in rats. Some data [23, 40, 41] suggest that postsynaptic rather than presynaptic 5-HT_1A_R is implicated in this effect in rats. Despite that these data further suggest the agonist-like profile of Zn at 5-HT_1A_R as in mice.

### Lower Lip Retraction and 5-HT Syndrome

Another behavioural approach used to characterize 5-HT_1A_R mediated activity is the induction of lower lip retraction (LLR). As shown by Berendsen et al. [20, 21], LLR was induced by the 8-OH-DPAT, buspirone, ipsapirone—agonists or partial agonists with a high affinity for 5-HT_1A_ binding sites in rat brain but not by serotonergic agents such as 5-MeO-DMT, mCPP or DOI with weaker affinity for 5-HT_1A_R but with high affinity for 5-HT_2A_R, 5-HT_2B/2C_R or 5-HT_2A/2C_R, respectively [20, 21]. The effect induced by 8-OH-DPAT was attenuated by 5-HT_1A_ antagonists such as WAY-100135 or WAY-100635 (which by themselves did not produce the LLR [42]), as well as by the above-mentioned nonselective serotonergic agents of moderate 5-HT_1A_R affinity which can act as antagonists of 5-HT_1A_R. In the case of location of receptors mediating this effect, it is suggested that LLR produced by the selective 5-HT_1A_R agonists has been attributed to autoreceptor activation [20, 21].

In our study, Zn did not induce LLR but significantly and dose-dependently blocked the LLR induced by 8-OH-DPAT. As was mentioned above, the LLR is specific only for compounds with high efficacy at 5-HT_1A_Rs; thus, the lack of this effect after Zn treatment with simultaneous blockade of 8-OH-DPAT action might result from the fact that Zn, like 5-MeO-DMT, mCPP or DOI, can also modulate other types of serotonin receptors, especially from 5-HT_2_R family. It was, for instance, found that chronic Zn administration increased the density of 5-HT_2A_ serotonin receptors in the frontal cortex [9]. Moreover, ritanserin, the 5-HT_2A/C_ receptor antagonist, blocked the antidepressant-like effect of Zn in the FST [10].

Another behavioural effect induced by 5-HT_1A_R agonists is serotonin syndrome that is believed to reflect postsynaptic 5-HT_1A_R activation. It is observed after higher doses of 8-OH-DPAT and is associated with the enhanced 5-HT synthesis [43]. In the behavioural syndrome tests, Zn given alone did not evoke flat body posture or forepaw trading, however, in the higher doses strongly inhibited both FBP and FT showing a postsynaptic 5-HT_1A_R antagonist-like profile.

Taken together, our data suggest that Zn has concentration-dependent actions in the above-mentioned behavioural responses that are mediated by 5-HT_1A_Rs. Thus, LLR and hypothermia are produced following administration of low doses of Zn or 8-OH-DPAT and are attributed selectively to autoreceptor activation. Interestingly, Zn induced hypothermia in mice at lower doses than in rats. Higher doses of Zn were needed to reverse 8-OH-DPAT induced LLR, and only higher doses of Zn blocked 8-OH-DPAT induced FBP/FT. These results are consistent with a potentiating effect of Zn on agonist actions at 5-HT_1A_R at low doses and an antagonist effect at higher doses, although the absolute dose depends on the animal model and behaviour studied.

### Forced Swim Test

The 5-HT_1A_R agonist 8-OH-DPAT and several others serotonin agonists with varying degrees of selectivity for different subtypes of 5-HTRs produce antidepressant-like behaviour in the FST. Wieland and Lucki, [44] showed that p-chlorophenylalanine (pCPA), which is known to reduce the concentration of serotonin in the brain by inhibiting its biosynthesis, did not block the antidepressant effect of 8-OH-DPAT in the FST in rats. These and other studies suggest that the antidepressant-like effects of 5-HT_1A_R agonists are mediated by postsynaptic 5-HT_1A_Rs [19, 44].

Zn also induces antidepressant-like effects in the FST both in rats and mice [10, 45–48]. Our earlier studies performed in mice [10] and the present studies in rats indicated that pretreatment with WAY-100635 blocked the effect of Zn. Furthermore, when Zn was given together with 8-OH-DPAT at the doses that were ineffective in the FST, it decreased immobility time, which suggests the additive effect of both compounds in this test and the agonist-like effect of Zn in the FST. Interestingly, the results of the 5-HT_1A_ autoreceptor KO mice in the FST demonstrated that the lack of presynaptic 5-HT_1A_Rs only partially blocked the anti-immobility effect induced by Zn in mice; however, pCPA pretreatment completely blocked the antidepressant-like effect of Zn in mice [10]. These data suggests that presynaptic receptors may be also implicated in the Zn-induced effects in the FST. These results also showed that Zn does not work without endogenous serotonin, which can be attributed to its allosteric mechanism of 5-HT_1A_R regulation because allosteric modulators may act only in conjunction with physiological receptor activation. It should be noted, however, that the allosteric nature of Zn described previously [13], which suggests only the inhibition of both agonist and antagonist interactions at 5-HT_1A_Rs, cannot explain the agonistic-like effects of Zn observed in our in vivo studies. In contrast, the current in vitro binding data, which showed characteristics of positive allosteric modulation in the presence of 10 μM of Zn and the inhibition of agonist action at sub-millimolar Zn concentrations, are more coherent with the results observed in vivo.

## Conclusions

In summary, our studies provide new data regarding the dual mechanism of Zn action at 5-HT_1A_Rs, which may underlie its antidepressant-like effects observed in the behavioural tests. The in vitro radioligand results revealed biphasic effects, involving allosteric potentiation of agonist binding at sub-micromolar Zn concentrations and inhibition at sub-millimolar Zn concentrations. Given the therapeutic potential of Zn, the in vitro results obtained for lower Zn concentration (10 μM) are within the range of reported extracellular free Zn concentrations (1–100 μM) and thus potentiating effects may be physiologically more relevant than inhibition observed using higher Zn concentrations at which toxic effects may be expected.

In behavioural paradigms, which are commonly used to distinguish the pharmacological profile of new compounds at 5-HT_1A_R, both agonist and antagonist-like effects of Zn at 5-HT_1A_Rs were found, and these data are consistent with results from the in vitro radioligand studies revealing biphasic Zn effects at 5-HT_1A_R, involving allosteric potentiation of agonist binding and inhibition. Taking into account results of in vivo studies with the use of wild-type and 5-HT_1A_ autoreceptor knockout animals, it seems that Zn can affect both pre- and postsynaptic 5-HT_1A_Rs. It should be, however, stressed that more studies are needed to explain this complex mechanism of antidepressant-like effect of Zn via modulation of serotonin system, especially that there are many controversies in the literature concerning the participation of the pre- and postsynaptic 5-HT_1A_Rs in the behavioural tests in rodents.

## Abbreviations

DOI: (±)-2,5-Dimethoxy-4-iodoamphetamine
FBP: Flat body posture
FST: Forced swim test
FT: Forepaw treading
GalR1: Galanin 1 receptor
HEK293: Human embryonic kidney 293
5-HT_1A_R: 5-HT_1A_ receptor
5-HT: 5-Hydroxytryptamine (serotonin)
5-MeO-DMT: 5-Methoxy-N,N-dimethyltryptamine
8-OH-DPAT: 8-Hydroxy-2-(di-n-propylamino)tetralin
LLR: Lower lip retraction
mCPP: 1-(3-Chlorophenyl)piperazine
pCPA: p-Chlorophenylalanine
PFC: Prefrontal cortex
WAY-100135: (S)-N-tert-Butyl-3-(4-(2-methoxyphenyl)-piperazin-1-yl)-2-phenylpropanamide dihydrochloride
WAY-100635: N-[2[4-(2-methoxyphenyl)-1-piperazinyl]ethyl]-N-(2-pyridyl)cyclohexanecarboxamide
